# Nuclear PKM2: a signal receiver, a gene programmer, and a metabolic modulator

**DOI:** 10.1186/s12929-025-01170-6

**Published:** 2025-08-11

**Authors:** Tsan-Jan Chen, Chun-Hsien Wu, Mien-Chie Hung, Wen-Ching Wang, Hsing-Jien Kung

**Affiliations:** 1 Institute of Molecular and Cellular Biology, College of Life Sciences and Medicine, National Tsing Hua University, Hsinchu, Taiwan; 2 Graduate Institute of Cancer Biology and Drug Discovery, College of Medical Science and Technology, Taipei Medical University, Taipei, Taiwan; 3 Graduate Institute of Biomedical Sciences and Center for Molecular Medicine, China Medical University, Taichung, Taiwan; 4 Institute of Biochemical Sciences, National Taiwan University, Taipei, Taiwan; 5 Department of Biochemistry and Molecular Medicine, Comprehensive Cancer Center, University of California, Davis, Sacramento, CA USA

**Keywords:** Nuclear PKM2, Nuclear translocation, Post-translational modification, Oncogenic signaling, Cancer metabolism, Signal receiver, Gene programmer, Metabolic modulator

## Abstract

Pyruvate kinase M2 (PKM2) is a key enzyme involved in glycolysis, yet its role in cancer extends far beyond metabolic flux. Unlike its isoform PKM1, PKM2 exhibits unique regulatory properties due to alternative splicing and dynamic structural plasticity, enabling it to translocate into the nucleus. Once nuclear, PKM2 functions as a signal receiver, gene programmer, and metabolic modulator by acting as a co-transcriptional activator and protein kinase. In this capacity, nPKM2 (nuclear PKM2) orchestrates the transcription of genes involved in glycolysis, lipogenesis, redox homeostasis, and cell cycle progression, thereby reinforcing the Warburg effect and promoting tumor growth, metastasis, and resistance to stress. In this regard, nPKM2 can be considered as the oncogenic component of PKM2. This review consolidates current knowledge on the structural basis of PKM2 assembly and the post-translational modifications that govern its oligomeric state and nuclear import. We also explore emerging therapeutic strategies aimed at targeting nPKM2, including small-molecule modulators that stabilize its cytosolic tetrameric form or disrupt its nuclear functions. Ultimately, the multifaceted roles of nuclear PKM2 underscore its significance as a critical oncoprotein and a promising target for precision cancer therapy.

## Introduction

Pyruvate kinase (PK) is the rate-limiting enzyme in the last step of glycolysis, catalyzing the conversion of phosphoenolpyruvate (PEP) and ADP into pyruvate and ATP, and playing a central role in cellular energy metabolism. Two alternatively spliced isoforms, PKM1 and PKM2, arise from mutually exclusive splicing of exons 9 and 10, with PKM1 forming a stable tetramer that exhibits high enzymatic activity and robustly supports oxidative phosphorylation. In contrast, PKM2, distinguished by a unique 56-amino acid segment encoded by exon 10, displays modulable activity in response to diverse stimuli through mechanisms including post-translational modifications (PTMs), metabolite bindings, and protein–protein interactions [91, 170, 177]. Notably, while PKM1 is a cytosolic form, PKM2 can translocate to the nucleus, where it functions as a transcriptional coactivator and protein kinase. This nuclear isoform (nPKM2) reprograms gene expression to promote oncogenic processes.

In recent years, PKM2 has received significant attention for its dual roles in metabolism and gene regulation, with many excellent reviews focusing on its contribution to the Warburg effect [106, 170]. A noticeable gap remains regarding the specific functions and therapeutic implications of nPKM2. Given the expanding body of literature, it is essential to consolidate current findings and highlight the emerging strategies for targeting nPKM2 in cancer [185]. This review aims to address that gap by first discussing the structural basis that enables PKM2 to undergo nuclear translocation and by elucidating how its activity is modulated by PTMs and protein–protein interactions. We then explore the oncogenic transcriptional programs driven by nPKM2, and finally, we examine how small-molecule modulators that target nPKM2 may disrupt tumor progression, underscoring nPKM2’s potential as both a biomarker and a therapeutic target in cancer.

## Mechanisms of PKM2 nuclear translocation

### Structural basis of PKM2 assembly, enzymatic activity, and nuclear translocation

**Fig. 1:**
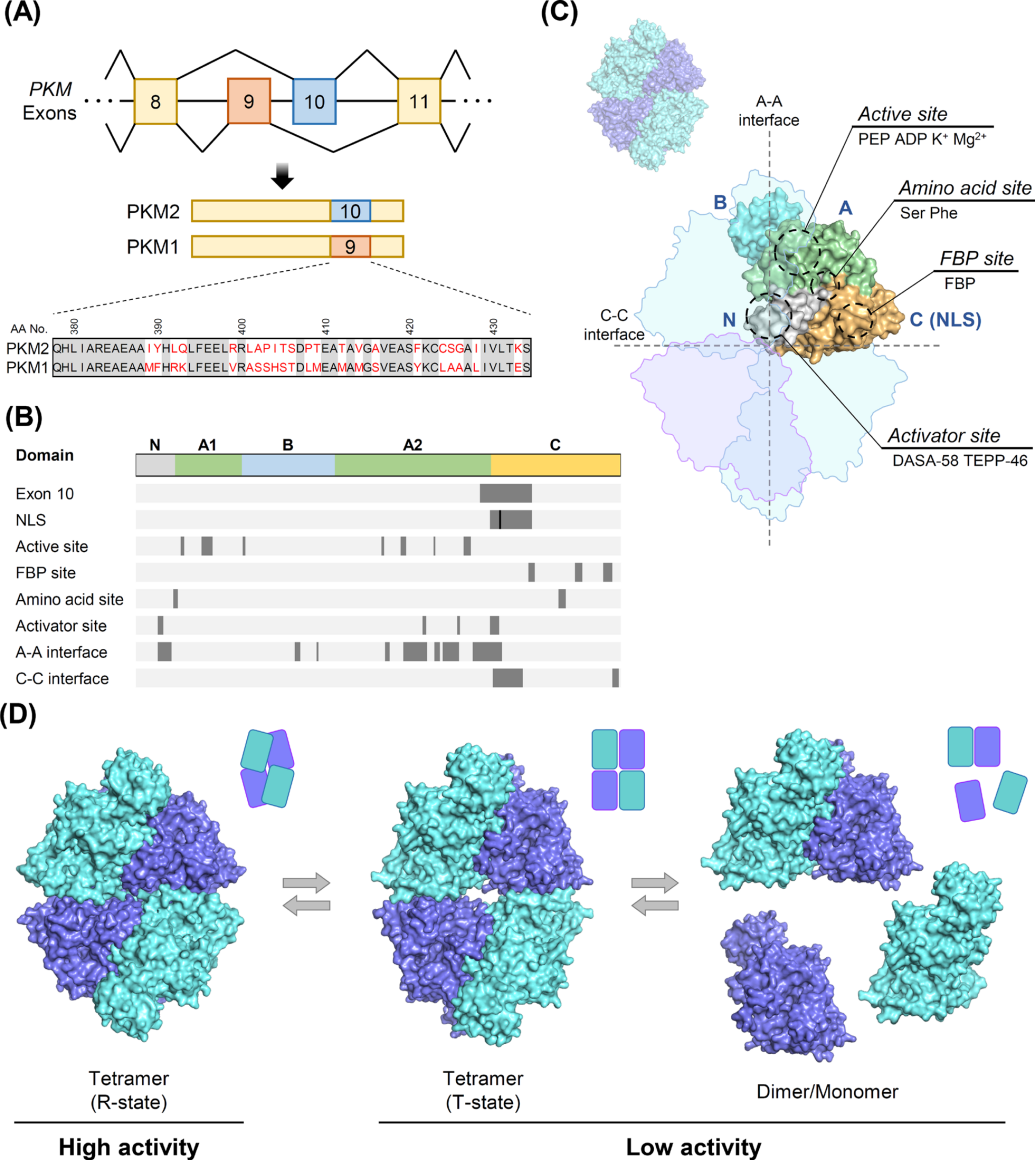
Structural organization and allosteric regulation of PKM2. **A** The *PKM* gene undergoes mutually exclusive alternative splicing, yielding two isoforms, PKM1 (exon 9) and PKM2 (exon 10). The sequence alignment below highlights the 56–amino acid difference encoded by exon 10 that underlies unique regulatory properties. **B** Linear domain map of PKM2: the **N** domain (residues 1–43), the **A** domain (**A1**: residues 44–116 and **A2**: residues 219–389), the **B** domain (residues 117–218), and the **C** domain (residues 390–531). Shaded ticks mark the exon 10 region, nuclear localization signal (NLS) with R399/R400 marked with a black stick, active-site, amino-acid, fructose-1,6-bisphosphate (FBP), and small-molecule activator (DASA-58/TEPP-46) binding sites, as well as the **A**–**A** and** C**–**C** interfaces. **C** Surface model of one PKM2 subunit colored by domain (**N**, gray; **A1**/**A2**, green; **B**, cyan; **C**, ochre). Key functional sites are labeled: active site (PEP·ADP·K^+^·Mg^2+^), amino-acid site (Ser/Phe), FBP site, and activator site (DASA-58/TEPP-46). Dashed lines indicate the **A**–**A** and **C**–**C** interfaces that mediate tetramer assembly. **D** Conformational equilibrium of PKM2: the high-activity R-state tetramer (left) and the low-activity T-state tetramer (center) interconvert with dissociated dimer/monomer forms (right). Endogenous metabolites (serine, SAICAR, butyrate) and synthetic activators (DASA-58, TEPP-46) modulate this equilibrium toward the R-state, thereby enhancing catalytic output and blocking the structural transitions required for PKM2’s nuclear import and transcriptional functions. The structural models are illustrated based on PDB models 3SRD and 4FXJ

PKM2’s primary metabolic function relies on a stable tetrameric assembly that conducts its pyruvate kinase activity, a process central to glucose metabolism and energy homeostasis [91]. In contrast to PKM1, which is generated by mutually exclusive alternative splicing of exon 9 and exists as a constitutive tetramer with high enzymatic activity, PKM2 includes exon 10 in its **C**-terminal domain near the dimer–dimer interface, endowing it with unique structural and regulatory properties [27]. Crystal structures of PKM2 show a tetramer composed of four subunits organized into four distinct domains: the **N** domain (residues 1–43); the **A** domain (subdivided into **A1**: residues 44–116 and **A2**: residues 219–389), which contains the active site for the phosphate transfer reaction; the **B** domain (residues 117–218), which bridges **A1** and **A2**; and the **C** domain (residues 390–531), which harbors regulatory elements including the fructose-1,6-bisphosphate (FBP) allosteric site near the **C**–**C** interface [34]. In the tetrameric conformation, extensive intersubunit contacts mask the nuclear localization signal (NLS) in the **C** domain, thereby channeling PKM2 toward its metabolic role in the cytosol (Fig. 1A‒B).

The dynamic assembly and enzymatic activity of PKM2 are regulated allosterically through key sites, including the FBP-binding site, an amino acid-binding site, and an activator site located at the **A**–**A** interface (Fig. 1C‒D). FBP serves as a strong activator that stabilizes the R-state of the tetramer, ensuring high catalytic output. A secondary allosteric site in a small cavity of the **A** domain near the **A**–**C** interface accommodates specific amino acids. For example, serine enhances PKM2 activity with moderate coupling to PEP, whereas phenylalanine shifts the enzyme to an inactive T-state [20, 93]. Additionally, metabolites such as SAICAR and butyrate modulate this dynamic assembly, enabling cancer cells to rapidly adapt their metabolism in response to changes in the microenvironmental cues [59, 75].

The nuclear import of PKM2 is closely tied to this oligomeric switch. The **C** domain contains the NLS, which is sequestered in the tetrameric state. Upon dissociation into dimeric or monomeric forms, the NLS becomes exposed, allowing PKM2 to bind importin α5 or nuclear proteins such as JMJD5/KDM8, facilitating its translocation into the nucleus [130, 158]. Once in the nucleus, PKM2 can act as a co-transcriptional activator for genes involved in tumor progression.

### PTM-driven conformational changes

**Fig. 2:**
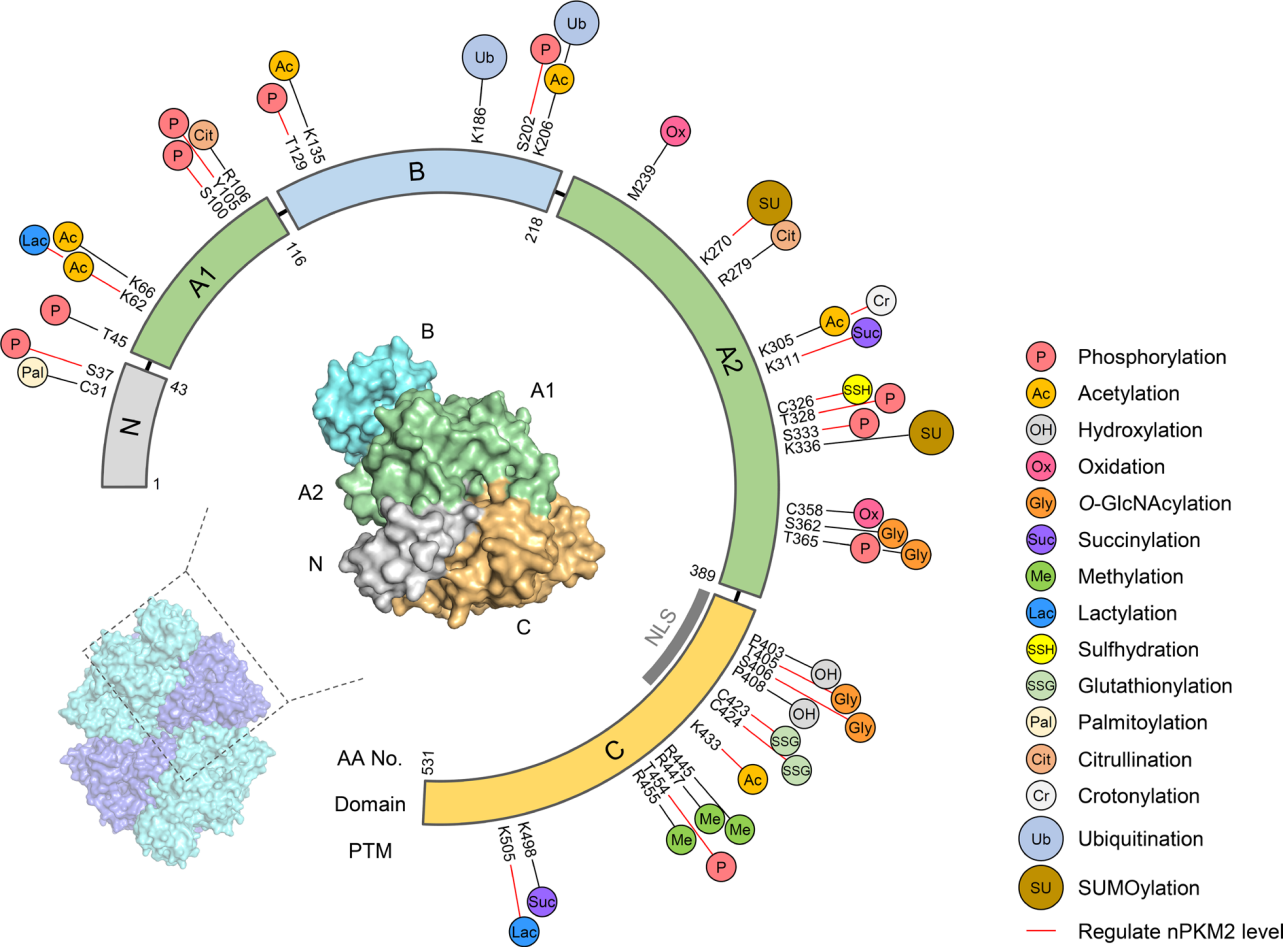
Post-translational modification landscape of PKM2. The circular schematic depicts PKM2’s primary structure (residues 1–531), color-coded by domain:** N** (gray), **A1** (green), **B** (cyan), **A2** (green), and **C** (ochre), with the nuclear localization signal (NLS) indicated in dark gray. Around the periphery, icons mark the positions and types of known PTMs: phosphorylation (P), acetylation (Ac), hydroxylation (OH), oxidation (Ox), *O*-GlcNAcylation (Gly), succinylation (Suc), methylation (Me), lactylation (Lac), sulfhydration (SSH), glutathionylation (SSG), palmitoylation (Pal), citrullination (Cit), crotonylation (Cr), ubiquitination (Ub), and SUMOylation (SU). Red connectors highlight those PTMs experimentally shown to regulate PKM2 nuclear import. The inset shows a PKM2 tetramer (surface view), with one subunit zoomed in to illustrate how PTMs cluster at key interdomain interfaces and regulatory sites

PTMs (post-translational modifications) act as molecular switches that reconfigure PKM2’s oligomeric state. In its tetrameric form, the NLS is masked by inter-subunit interactions. However, various PTMs promote a structural shift to dimeric or monomeric forms that expose the NLS, enabling PKM2 to engage the nuclear import factor and translocate into the nucleus, where it executes non-metabolic functions. Table 1 and Fig. 2 summarize the known PTM sites and their effects on PKM2 assembly.

**Table 1:** The PTMs of PKM2

PKM2 residue	PTM	Modifying enzyme/chemical		Level change when the PTM is added			Refs
		Modifier	De-modifier	PK Activity	Tetramer	nPKM2 Level	
C31	Palmitoylation	zDHHC13		Decrease	Decrease	–	[44]
S37	Phosphorylation	ERK CDK6-cyclin D3 KHK-A PKCε	Cdc25A	Decrease	Decrease	Increase	[6, 65, 78, 102, 128, 158]
T45	Phosphorylation	Aurora B		–	–	–	[54]
K62	Lactylation			Increase	Increase	Decrease	[132]
K62	Acetylation		HDAC8	–	–	Decrease	[169]
K66	Acetylation	GCN5		–	–	–	[171]
S100	Phosphorylation	Chk2		–	–	Decrease	[161]
Y105	Phosphorylation	FGFR1 Syk ErbB2 NPM-ALK PKCε	PTP1B SHP-1	Decrease	Decrease	Increase	[10, 28, 50, 65, 116, 122, 178, 184, 186]
R106/R279	Citrullination	PADI1/PADI3		Increase	–	–	[32]
T129	Phosphorylation	eEF2K		–	Increase	Decrease	[149]
K135/K206	Acetylation		SIRT1	Increase	Increase	–	[76]
K186/K206	Ubiquitination	Parkin		Decrease	–	–	[82]
S202	Phosphorylation	AKT1		–	–	Increase	[101]
M239	Oxidation		MSRA	Increase	Increase	–	[189]
K270	SUMOylation	PIAS3		Decrease	Decrease	Increase	[146]
K305	Acetylation	PCAF	HDAC SIRT2	Decrease	Decrease	–	[88, 100]
K305	Crotonylation			Increase	–	Increase	[16]
K311	Succinylation		SIRT5	Decrease	Decrease	Increase	[129]
C326	Sulfhydration	H_2_S		Decrease	Decrease	Increase	[133]
T328	Phosphorylation	GSK3β ATM		–	–	Increase (Retention)	[53, 117]
S333	Phosphorylation	ULK1		–	Increase	Decrease	[187]
K336	SUMOylation	SAE1/UBA2		–	–	–	[127]
C358	Oxidation	ROS		Decrease	Decrease	–	[2]
S362/T365	*O*-GlcNAcylation	OGT		Decrease	Decrease	–	[116]
T365	Phosphorylation	JNK1		Increase	–	–	[51]
P403/P408	Hydroxylation	PHD3		–	–	–	[87]
T405/S406	*O*-GlcNAcylation	OGT		Decrease	Decrease	Increase	[80]
C423/424	Glutathionylation	ROS		–	–	Increase	[143]
K433	Acetylation	p300	SIRT6	Decrease	Decrease	Increase	[11, 89]
R445/R447/R455	Methylation	CARM1		–	–	–	[80]
T454	Phosphorylation	PIM2		Decrease	–	Increase	[162, 163]
K498	Succinylation		SIRT5	Increase	–	–	[148]
K505	Lactylation			–	–	Increase	[104]
Unknown site(s)	Phosphorylation	SphK1				Increase	[76]
	Ubiquitination	TRIM21 TRIM33				Increase (Retention)	[152, 153]
	Ubiquitination	Laforin/Malin	PSMD14	Increase	Increase	Decrease	[121, 124]
	Acetylation	OGA					[116]
	O-GlcNAcylation	OGT	OGA	Decrease			[19]
	SUMOylation	Ubc9					[134]
	Lactylation	p300					[76]

Phosphorylation is one of the most extensively studied PTMs influencing PKM2. Phosphorylation at S37, mediated by ERK1/2 [158] or Y105 by kinases such as FGFR1/ALK [184] and Syk [28], disrupts tetrameric interactions and favors dimer formation. In particular, S37 phosphorylation, followed by a conformational isomerization mediated by PIN1, shifts PKM2 to a dimeric form with an exposed NLS [158]. Acetylation at lysine residues K305 and K433, catalyzed by acetyltransferases such as PCAF, p300, and TIP60, similarly destabilizes the tetrameric interface, allowing PKM2 to “open up” for nuclear import [88, 89]. SUMOylation at K336 by PIAS3 further promotes a dimeric conformation and may act in concert with phosphorylations to block activator binding (FBP) [98, 127].

Other PTMs contribute significantly to the dynamic assembly of PKM2. Modifications such as *O*-GlcNAcylation at T405/S406, certain ubiquitination patterns, succinylation, sulfhydration, and lactylation have all been reported to influence PKM2 assembly (see Table 1). Notably, lactylation at K505 of PKM2 promotes its nuclear translocation in hepatocellular carcinoma under high glucose conditions [104]. In addition, PKM2 harbors a SUMO-binding motif (IKII, residues 265–268) that can bind SUMO non-covalently, potentially affecting both its oligomeric state and enhancing nuclear retention [98, 127, 146]. Emerging evidence further indicates that citrullination can reprogram cross-talk between PKM2 ligands to regulate glycolysis [32]. Furthermore, binding of poly(ADP-ribose) (PAR) helps anchor PKM2 within the nucleus, expanding the repertoire of mechanisms controlling PKM2 subcellular localization [71]. Lysine crotonylation at PKM2 K305 facilitates the nuclear translocation of PKM2 and promotes aerobic glycolysis in vascular smooth muscle cells [16].

Collectively, these PTMs shape PKM2’s conformational landscape and functional outcome, switching the enzyme from a high-activity tetramer that catalyzes glycolysis in the cytosol to a nuclear-prone form that can act as a coactivator for oncogenic gene expression. By dynamically regulating PKM2’s assembly and nuclear import, PTMs enable cancer cells to fine-tune metabolic flux and transcriptional programs to meet the demands of rapid proliferation and adaptation.

## Protein mediators of nuclear translocation

There are a large number of proteins whose direct interactions with PKM2 facilitate PKM2’s nuclear translocation. These factors serve as signal transducers to reprogram the genome via nPKM2 in response to the external environment. Some of them, which are overexpressed in cancer cells, are oncogenes and potential targets for intervention. There are also factors that suppress such a translocation. In this section and Table 2, we summarize nPKM2 interacting proteins: 1) directly translocate PKM2 by the recruitment of importins, 2) assemble or recruit enzymes that catalyze PTMs of PKM2, and 3) facilitate nuclear exit of PKM2.

**Table 2:** Protein mediators of PKM2 nuclear translocation

Interacting protein	Mechanism of nuclear translocation and retention	References
Inducer of nuclear translocation		
Importin complex		
KDM8/JMJD5	KDM8 binds PKM2 at the dimer interface to block tetramer formation and provides an NLS for translocation	[130]
DDX39B	DDX39B stabilizes PKM2 and recruits importin α5 to accelerate the nuclear translocation of PKM2 independent of ERK1/2-mediated phosphorylation of PKM2	[179]
FOXM1D	FOXM1D recruits importin-4 and forms a complex with PKM2 and NF-κB to translocate PKM2	[172]
AMPK	AMPK and PKM2 are translocated to the nucleus by a Ran-mediated mechanism	[23, 83, 186]
Phosphorylation		
ERK2	ERK2 binds Ile 429/Leu 431 of PKM2 to phosphorylate S37, which binds importin α5 after PIN1 isomerization	[155, 158]
Rack1	Rack1 connects FGFR and PKM2 to phosphorylate PKM2 at Y105	[184]
Syk	Syk directly phosphorylates PKM2	[28]
EGFRvIII	EGFRwt/vIII binds PKM2 and translocates PKM2 into the nucleus	[57, 167]
Cdc25A	Cdc25a binds PKM2 in the nucleus to dephosphorylate PKM2 at S37 to activate p H3T11 and H3K9Ac in ErbB2 promoter	[128]
SphK1	SphK1 phosphorylates PKM2	[76]
KHK-A	KHK-A phosphorylates PKM2 and facilitates its binding to importin α5	[102]
MYG1	MYG1 recruits HSP90/GSK3β to bind and phosphorylate PKM2 at T328	[53]
ATM	ATM phosphorylates PKM2 at T328	[117]
AKT	AKT phosphorylates PKM2 at S202	[101]
PPP1R26	PPP1R26 binds Ser37-phosphorylated PKM2 and retains PKM2 in the nucleus	[159]
ATP6V0C	ATP6V0C binds PKM2 with increased Y105 phosphorylation	[118]
PP4R1	PP4R1 recruits ERK1/2 to bind PKM2 to promote phosphorylation of PKM2	[29]
PARP1	PARP1 activates PKM2 nuclear translocation, evidenced by increasing pY105 PKM2	[114]
SUMO and ubiquitination		
SAE1/UBA2	SAE1/UBA2 SUMOylates PKM2 at K336 to promote its phosphorylation	[127]
CHAC1	CHAC1 recruits UBA2 to bind and SUMOylate PKM2	[98]
ESM1	ESM1 recruits UBA2 to bind and SUMOylate PKM2	[165]
GTPBP4	GTPBP4 recruits UBA2 to induce SUMO-1 binding to PKM2 at IKII (265–268) and dimerization of PKM2	[185]
TRIM33	TRIM33 binds and ubiquitinates nPKM2 with K63-ubiquitination in the nucleus, which retains PKM2 in the nucleus	[153]
PSMD14	PSMD14 decreased K63-linked ubiquitination on PKM2 to induce dimers/monomers and nuclear translocation of PKM2	[121]
Acetylation and succinylation		
SIRT5	SIRT5 succinylates PKM2 at K311 to promote entry into the nucleus	[129]
PSAT1	PSAT1 binds PKM2 and is associated with acetylation at K433	[13]
HDAC8	HDAC8 deacetylates PKM2 K62 to induce PKM2 nuclear translocation	[169]
Mechanism unclear		
PDIA3	PDIA3 translocates PKM2 in a phosphorylation-independent way	[151]
MTHD1	MTDH/PKM2/β-catenin complex inhibits PKM2 tetramer formation	[29]
TRIM21	TRIM21 promotes PKM2 nuclear translocation	[152]
Fgl2	Fgl2 facilitated PKM2 nuclear translocation	[46]
SP100A	SP100A binds ERK1/2-PKM2-importin complex to translocate into the nucleus	[35]
hnRNPF and Lnc-HIFAL	HIFAL RNA assembles PKM2/PHD3 complex into the nucleus via binding hnRNPF to enhance HIF-1α transactivation	[183]
TR3	TR3 protects nuclear PKM2 from degradation	[81]
Shikonin	Shikonin induces the nuclear translocation of PKM2 for recruiting NRF2	[85]
ATF2	ATF2 binds nuclear PKM2 and is phsophorylated by PKM2	[70]
TBC1D8	TBC1D8 hinders tetramer formation	[25]
Suppressor of nuclear translocation		
Vitamin B5/Coenzyme A	Coenzyme A binds PKM2 to impede its phosphorylation and nuclear translocation	[24]
Chk2	Chk2 promotes the nuclear export of PKM2 by phosphorylating PKM2 at Ser100	[161]
EXT1	EXT1 facilitates PKM2 to exit the nucleus	[53, 76]
SIRT1	SIRT1 suppresses PKM2 nuclear translocation	[24]
JOSD2	JOSD2 inhibits nuclear localization of PKM2 by reducing its K433 acetylation modification	[66]
SIRT6	SIRT6 deacetylates nuclear PKM2 at K433 to suppress its nuclear localization	[11]
Exportin 4	Exportin 4 mediates the nuclear export of PKM2	[11]
LA	LA decreases tyrosine phosphorylation Y105 on PKM2 and nuclear translocation	[97]
SAA	SAA directly interacts with PKM2 at its activator pocket, inhibiting phosphorylation of Y105	[188]
Oridonin	Oridonin reduces importin α5 binding to the PKM2 dimer	[21]
ART	ART induces lactylation of PKM2	[76]
TRIM35	TRIM35 (E3 ligase)-mediated degradation of nuclear PKM2	[84]
BLM	BLM inhibits dimer formation and nuclear translocation of PKM2	[18]

### Proteins associated with the importin complex

Importin α5, which forms a complex with importin β, is involved in nuclear transport. JMJD5/KDM8, a histone demethylase overexpressed in prostate and breast cancers, binds PKM2 at the dimer–dimer interface in favor of dimer formation and provides its own nuclear localization signal to recruit importin to facilitate the translocation [130, 131]. DDX39B, an RNA helicase, recruits importin α5 to the PKM2 complex and translocates PKM2 in a phosphorylation-independent fashion [179], as does PDIA3, an isomerase [151]. The Ran GTPase, a critical regulator of nuclear transport, has also been shown to assist in shuttling PKM2-containing complexes into the nucleus [186].

### Proteins involved in phosphorylation

As described above, PTMs such as phosphorylation, SUMOylation, ubiquitination, and acetylation were reported to favor dimer formation and expose importin binding (Table 1). Yang et al. [158] showed that ERK2 phosphorylates PKM2 at S37, which, upon isomerization by PIN1, is able to bind importin α5 to translocate PKM2 to the nucleus. Other kinases, including Syk [28], FGFR [186], EGFRvIII [57, 167], ErbB2 [186], SphK1 [53, 76], KHK [102], GSK3β [53], ATM [117], AKT [101], are found to be binders and inducers for nuclear translocation of PKM2 (Table 2). In most cases, S37 and Y105 of PKM2 are phosphorylation sites related to translocation. However, GSK3β and ATM target T328 of PKM2 [53, 117], whereas AKT phosphorylates T202 [101], all with the same consequence. Interestingly, Cdc25A, a phosphatase, also interacts with PKM2 to dephosphorylate S37, enabling the activation of ErbB2 and c-Myc [78]. The detailed mechanism requires further investigation. PPP1R26 binds pS37 to retain PKM2 in the nucleus. Vacuolar H^+^-adenosine triphosphatase (ATPase) subunit V0C (ATP6V0C) interacts with PKM2 to gain Y105 phosphorylation and facilitates nuclear translocation [118]. However, not all phosphorylation leads to nuclear retention; for instance, eEF2K phosphorylates PKM2 at T129 to reduce dimer formation [149].

### Proteins involved in SUMOylation and ubiquitination

SAE1/UBA2, the sole SUMO activating enzyme in cells, binds and SUMOylates PKM2 at K336, favoring nuclear translocation by providing a scaffold for additional phosphorylation events that deflect the binding of agonist FBP [127]. Proteins such as CHAC1 [98], ESM1 [165], or GTPBP4 [185] that interact with PKM2 and recruit UBA2 to induce SUMOylation or SUMO-1 binding also facilitate nuclear translocation. The situation of ubiquitination is more complex. TRIM33, a Ub E3 ligase, binds and ubiquitinates PKM2 with K63-ubiquitination to retain PKM2 in the nucleus [153], while PSMD14 [121], a deubiquitinase, reduces K63-linked ubiquitination to favor dimer formation.

### Proteins involved in succinylation and acetylation

It was first shown by Lv et al. [89] that EGF stimulates acetylation of PKM2 at K433, which blocks the binding of FBP, an agonist of tetramer formation, thereby enhancing the nuclear translocation of PKM2. SIRT5 is a deacetylase that has the dual function of inducing succinylation of PKM2 at K311 to disengage the tetramer into a dimer [129], thereby facilitating PKM2’s nuclear translocation. PSAT1 (phosphoserine aminotransferase) binds PKM2 to translocate into the nucleus, which is dependent on K433 acetylation [13]. Interestingly, not all acetylations send PKM2 to the nucleus. For instance, HDAC8 binds PKM2 and deacetylates K62 to facilitate transport [169].

### Proteins involved in suppressing PKM2 nuclear translocation

Above, we discussed proteins that enhance the nuclear translocation of PKM2. There are also PKM2 binding proteins that suppress such translocation. For instance, Chk2 phosphorylates PKM2 at S100 and promotes nuclear exit [161], and EXT1 (exostosin-1) hastens the exit of PKM2 to the cytosol [74]. SIRT1 and SIRT2 deacetylases [24], as well as JOSD2 [66], a deubiquitinase, inhibit PKM2 nuclear translocation by deacetylation. SIRT6 specifically deacetylates PKM2 at K433 and, via exportin 4, facilitates its export from the nucleus [11]. Small SHP-1 tyrosine phosphatase [122] and small molecules such as LA and SAA suppress tyrosine phosphorylation of Y105, and oridonin reduces importin α5 binding, and ART induces acetylation [136], all hindering nuclear translocation of PKM2. The latter small molecules are potential targeting agents for nuclear PKM2-associated diseases. eEF2K phosphorylates PKM2 at T129 to prevent dimer formation [149]. TSC22D2 also reduces the level of nuclear PKM2, with a yet-to-be-determined mechanism [77].

Collectively, these diverse protein mediators and PTMs establish a finely tuned regulatory network that controls the nuclear translocation of PKM2. Understanding these interactions not only illuminates the molecular underpinnings of PKM2’s dual role in metabolism and gene regulation but also reveals potential targets for therapeutic intervention in cancers characterized by aberrant nPKM2 activity.

## RNA mediators of PKM2 nuclear translocation

In addition to protein–protein interaction, several long-non-coding RNAs (lncRNAs) play crucial roles in regulating the nuclear translocation of PKM2. MNX1-AS1, which is frequently overexpressed in hepatocellular carcinoma-derived cell lines and tissues, functions as a molecular scaffold that promotes the interaction between PKM2 and importin α5 [144]. In response to EGFR activation, the formation of this ternary complex facilitates the nuclear import of PKM2. HIFAL, a hypoxia-induced lncRNA, forms a complex with PKM2 and PHD3 under low oxygen conditions, driving PKM2 into the nucleus [183]. AC020978, another lncRNA upregulated under glucose starvation and hypoxia, is directly transactivated by HIF-1α. It interacts with and stabilizes PKM2, promoting the nuclear translocation of PKM2 and enhancing HIF-1α transcription activity [47]. Additionally, FEZF1-AS1, one of the highly overexpressed lncRNAs in colorectal cancer, binds and stabilizes PKM2, resulting in increased cytoplasmic and nuclear PKM2 levels [12]. Together, these lncRNAs serve as important RNA mediators that regulate PKM2’s subcellular localization and contribute to its role in oncogenic gene programming.

## PKM2 mutations involved in nuclear translocation

**Fig. 3:**
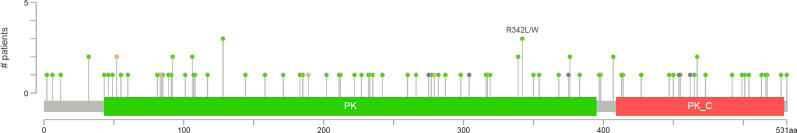
Sporadic PKM2 mutations in the exon 10 region and their association with oligomeric assembly. This schematic illustrates the domain architecture of PKM2 (residues 1–531), highlighting the core pyruvate kinase (PK) domain (green) and the PK_C domain (red). Each mutation from TCGA data, with several mutations clustering within the exon 10 region that is clinically relevant for allosteric regulation and oligomerization (https://www.cbioportal.org/)

Recent analyses of The Cancer Genome Atlas (TCGA) reveal that PKM2 harbors a spectrum of sporadic, scattered mutations rather than a single recurrent hotspot. Many of these mutations cluster in the exon 10 region and the **C**–**C** interface, areas critical for PKM2’s oligomeric assembly and allosteric regulation (Fig. 3). Notable exon 10 mutations such as H391Y, R399E, and G415R have been associated with various disease states. For example, the H391Y variant, originally identified in a patient with Bloom syndrome, affects PKM2’s cross-monomer interactions, driving tumorigenesis [5, 52]. These mutations lead to reduced allostery and increased nuclear translocation of PKM2 [27]. Moreover, the mutated forms of PKM2 tend to exhibit enhanced stability and stronger binding to nuclear mediators such as KDM8 and HIF-1α, thereby contributing to metabolic flexibility and aggressive tumor behavior.

## nPKM2 as a signal receiver

**Fig. 4:**
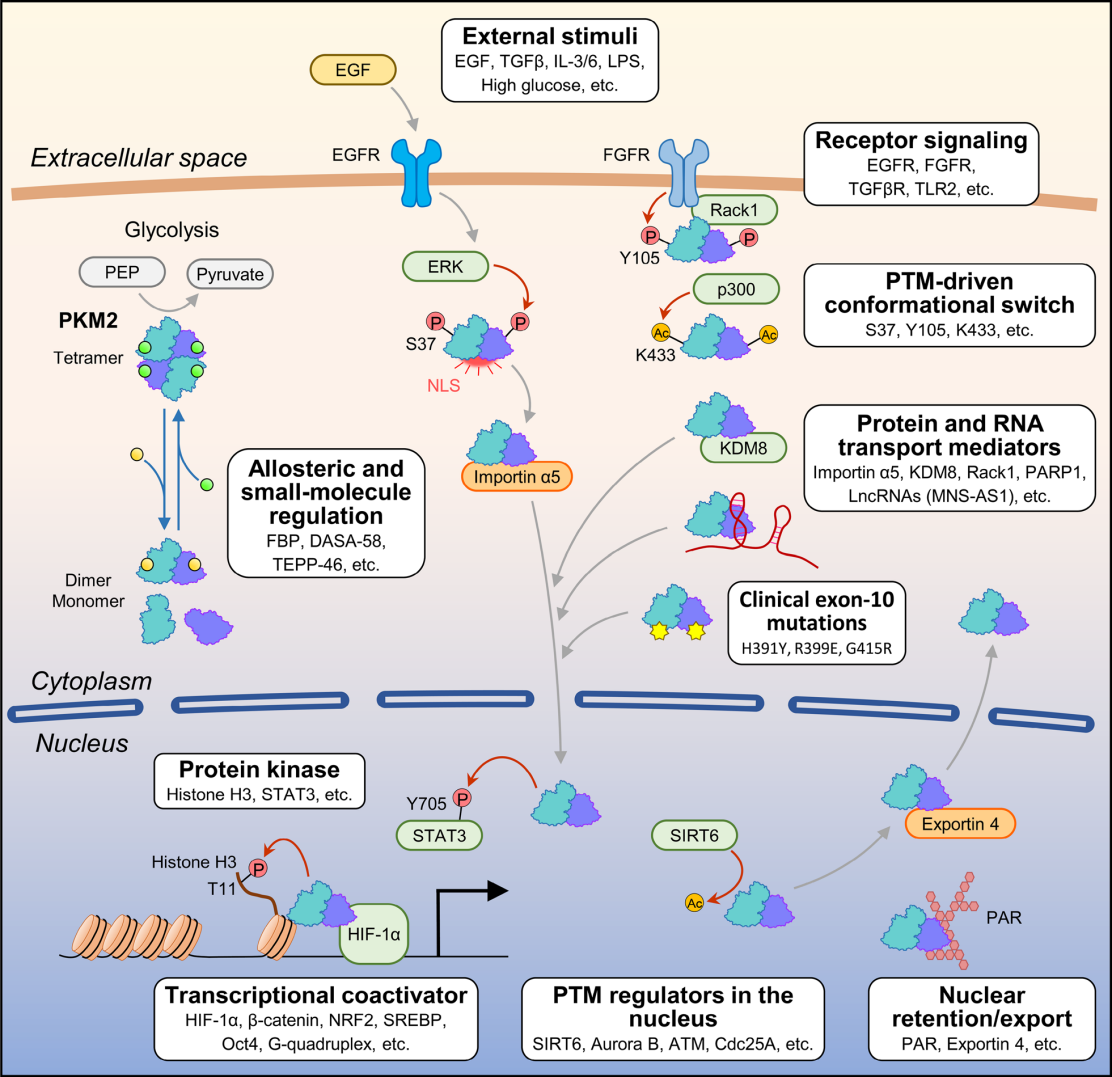
Integrated model of PKM2 nuclear translocation and nuclear functions. The schematic diagram illustrates how PKM2 shuttles between the cytosol and nucleus in response to metabolic cues, post-translational modifications (PTMs), and protein- or RNA-mediated interactions

Given the very large number of PTMs targeting PKM2, it is conceivable that nPKM2 has evolved to respond to extracellular stimuli to allow cancer cells to adapt to the ever-changing tumor micro-environment. Indeed, in breast cancer cell model, it was found that nPKM2 is critical for the survival of cancer cells, but not for their normal counterparts [186]. As shown in Table 3 and Fig. 4, growth stimuli, either in the form of soluble ligands or metabolites, are triggers of nuclear translocation events. Among these, growth factors that utilize receptor tyrosine kinases such as EGF [36, 89, 96, 156, 158], IGF1 [110], insulin [81], and HGF [33] are the predominant members. Oncogenic variant EGFRvIII, which is constitutively active and independent of EGF binding, also interacts with PKM2 [57, 167]. The seminal work of Yang et al. [158] demonstrated that EGF/EGFR activates ERK to phosphorylate PKM2 at S37 to provide a platform for importin binding to facilitate translocation, which is validated by other publications and extends to other ligands capable of activating ERK. Lv et al. [89] reported that EGF can also induce PKM2 nuclear translocation by p300-mediated acetylation at K433. On the other hand, erbB2/HER2 [186] and FGFR [184] induce tyrosine phosphorylation at Y105 to translocate PKM2. TGFβ sends the signal to translocated PKM2 to induce EMT [43] and PD-L1 expression [147]. Estrogen induces either Y105 [108] or S37 phosphorylation [169] of PKM2 for translocation. For cytokines, both IL-3 [45] and IL-6 [90] activate Jak2 to enhance PKM2 translocation. IL-6 does so by activating SHMT2, which reduces serine levels, thereby favoring dimer formation [90]. T cell activation [4, 15] also relies on the Y105 and S37 phosphorylation of PKM2 to achieve its goal.

**Table 3:** Signals involved in PKM2 nuclear translocation

Signal initiator	Signal pathways	References
Growth factor/Receptor tyrosine kinase		
EGF	nPKM2 phosphorylates H3T11 to activate genes in glioblastoma	[156]
	Activated ERK2 binds and phosphorylates PKM2 at S37 to provide an importin binding platform in glioblastoma cells	[158]
	S37 phosphorylation of PKM2	[96]
	S37 phosphorylation of PKM2 to induce DKK1 expression in HCC	[96]
	ERK-mediated PKM2 translocation to activate β-catenin in HCC	[36]
	p300-mediated K433 acetylation of PKM2 to block FBP binding in breast cancer cells	[89]
	PKM2 nuclear translocation in NPC and HNSCC, blocked by cetuximab	[26]
IGF1	Activated AKT mediates phosphorylation and dimerization of PKM2 to activate STAT3	[110]
Insulin	Stabilizes PKM2 by enhancing PKM2/TR3 interaction in HCC	[81]
HGF/MET	Activated ERK mediates S37 phosphorylation of PKM2 in retinoblastoma	[33]
EGFRwt/vIII	EGFRwt/vIII induces PKM2 translocation to activate β-catenin in HNSCC cells	[57]
EGFRvIII	Nuclear EGFRvIII induces PKM2 translocation and STAT3 phosphorylation in glioblastoma cells	[167]
EGF/EGFR	nPKM2 interacts with c-Src phosphorylated β-catenin	[157]
ErbB2	ErbB2 induces Y105 phosphorylation of PKM2 to enhance PKM2 and YAP nuclear localization for breast cancer stem cells	[186]
FGFR	RACK1 forms complex with FGFR and PKM2 to induce Y105 phosphorylation of PKM2	[184]
Growth factors	PKM2 dimer phosphorylates STAT3 Y705 in colon cancer cells	[38]
TGFβ		
TGFβ	TGFβ + EGF induce PKM2 translocation via SMAD2 and ERK and binding to TGIF2/HDAC3 to suppress CDH1 expression and activate EMT in colon cancer cells	[43]
TGFβ	Enriched nPKM2 promotes PD-L1 expression in tumor-associated macrophages	[147]
TGFβRII	Enriched nPKM2 regulates glucose metabolism in oral cancer-associated fibroblasts	[138]
Nuclear receptor		
Estradiol-17β (E2)	E2 induces Y105, S37 phosphorylation, oxidation, and translocation of PKM2 which binds ER for transcription in primary endometrial stromal cells	[109]
Estrogen	Estrogen activates mTOR to induce S37 phosphorylation of PKM2 in lymphangioleiomyomatosis	[86]
Cytokines and soluble factors		
IL-3	Jak2 activation to induce PKM2 nuclear translocation proliferation of Ba/F3 cell	[45]
IL-6	IL-6 activates Jak2/STAT3 to induce SHMT2 expression which reduce serine to increase PKM2 dimer formation	[90]
Oxidized LDL	Oxidized LDL induces Y105 phosphorylation of PKM2 to enhance PKM2-SREBP1 interaction	[62]
LPS	LPS activates DC cells with JNK activation and PKM2 acetylation to induce IL12p35	[56]
Hyaronan/CD44	HA/CD44 activates ERK (Thr202/Tyr204) phosphorylation to induce PKM2 translocation in HCC	[68]
Somatostatin (TT-232)	nPKM2 translocation with associated cell death in COS7 cells	[120]
TLR2	TLR2 activation induces PKM2 nuclear translocation in rheumatoid arthritis	[92]
Immune activation		
TCR + CD43	TCR + CD43 costimulation induces phosphorylation of PKM2 (Y105)/ STAT3 and MEK5/ERK5 in T cell hybridoma	[15]
CD3/CD28 activation	Induces Y105 and S37 phosphorylation of PKM2 in CD4 + T cells	[4]
Metabolites and chemicals		
Lactate	Lactate induces PKM2/STAT3 translocation to enhance IL-17 expression in CD4 + T cells	[103]
Valine	Valine activates TAS1R1-mTOR-DDX39B signal pathway to translocate PKM2 in BMEC	[17]
Nitric oxide	Nitric oxide induces PKM2 nuclear translocation via EGFR/ERK2 signaling to regulate glycolysis in ovarian cancer cells	[69]
Melatonin	Promotes nuclear translocation of PKM2 to bind NRF2 to suppress ferroptosis in HT-22 neuronal cells	[105]
High glucose	High glucose induces lactate and PKM2 nuclear translocation through lactylation at K505 in HCC	[104]
High glucose	High glucose induces Y105 phosphorylation of PKM2 in diabetes	[169]
Glucose deprivation	Activated AMPK translocates with PKM2 to the nucleus by Ran in human pancreatic and pulmonary adenocarcinoma cells	[186]
PCB126	PCB increases nuclear PKM2 in HCC	[119]
Polychlorinated biphenyls (PCBs)	Pollutant induces PKM2 nuclear translocation	[175]
CCl_4_	Induces PKM2 nuclear translocation to activate STAT3 in liver regeneration	[137]
Benzene	1,4-benzoquinone (1,4-BQ) induces acetylation of PKM2 at K66 in hematopoietic cells	[171]
OTA (ochratoxin A)	OTA induces S37 phosphorylation of PKM2	[135]
Metformin	Under glucose deprivation conditions, metformin treatment in renal carcinoma cells promotes nuclear AMPK binding to PKM2/ β-catenin	[83]
Oxaliplatin	OXA induces PKM2 translocation in OXA-sensitive colon cancer cells via activation of BMF, a death-related gene	[39]
Others		
Ionization radiation (IR)	IR induces PKM2 translocation in breast cancer stem cells	[166]

In addition to proteins and other macromolecules, PKM2 can also respond to external metabolites and chemicals. Thus, lactate, valine, nitric oxide, and melatonin are all found to trigger the nuclear translocation of PKM2 [17, 69, 83, 103]. The case of glucose is interesting as either high glucose or glucose depletion can trigger PKM2 translocation in different cell contexts. High glucose tends to generate a high level of lactate, which, through lactylation, is capable of translocating PKM2 [48, 104]. On the other hand, glucose depletion induces AMPK, which directly interacts with PKM2 for nuclear translocation [113, 186]. Finally, it is noteworthy that pollutants such as PCBs and toxic chemicals such as CCl_4_, benzene, and ochratoxin are also nuclear PKM2 inducers [39, 119, 135, 137, 171, 175].

Overall, nPKM2 generally plays a pathogenic role in enhancing cancer cell growth, EMT, and immune escape. It is most commonly triggered by oncogenic signals such as the aberrantly activated tyrosine kinases and serine kinases. Targeting PKM2 nuclear translocation remains a suitable anticancer strategy (see next section).

## nPKM2 as a gene programmer and metabolic modulator

Once translocated into the nucleus, PKM2 functions as a co-activator, interacting with key transcriptional factors and chromatin regulators to regulate genes involved in various aspects of metabolism (Table 4).

**Table 4:** nPKM2 as a gene programmer

Transcription mediators	Target genes/Biological output	References
HIF-1α	Aerobic glycolysis genes and HIF-1α target genes (GLUT1, GLUT3) to affect glycolysis	[60, 130, 173, 183]
	CCL2 and ischemia-induced neuroinflammation genes	[37]
	PFKFB3 to affect glycolysis	[99]
YAP	GLUT3	[64]
	ErbB2	[186]
SREBP	SREBP target genes and FASN	[62, 180]
EZH2	SLC16A9 to affect fatty acid β-oxidation	[176]
p65	VEGF	[8]
	IL-6, IL-8, and other inflammatory genes	[40]
NRF2	GPX4, SLC9A11, and BAG3	[85, 105]
β-catenin	CCND1, cyclin D1, HDAC3, via H3 acetylation	[157, 169]
H3	CCND1, MYC via H3pT11, H3K9Ac, and H3 acetylation	[17, 156]
Oct4	Stemness-related genes	[186]
STAT3	MEK5	[38]
	IL-6 and IL-1β	[115]
	TNF-α and IL-1β	[154]
	c-Myc	[149]
Folded G-quadruplexes	EMT genes (E to hyper EMT)	[1]
GATA4/6 & MDM2	Protection of GATA4/6 from cleavage MDM2-mediated degradation of p53	[84]

### Genes associated with glycolysis metabolism

HIF-1α is a master regulator of the Warburg effect, shifting cancer cells towards aerobic glycolysis [112]. In the nucleus, PKM2 amplifies the HIF-1α transcriptional program by directly binds HIF-1α, recruiting p300, and phosphorylating histone H3 at T11 to open up chromatin at hypoxia response elements (HREs), increasing the expression of glycolytic genes including LDHA, GLUT1, and GLUT3 [87, 123]. It also enhances the binding of HIF-1α and p300 to the HRE residing in the promoter of PFKFB3 [99]. Likewise, KDM8 carries PKM2 to the nucleus to form a ternary complex with HIF-1α, which is recruited to the promoters of *LDHA* and *PKM* to sustain the glycolytic pathway [99, 130]. Several reports relate YAP to PKM2-mediated HIF-1α activation. Under hypoxia conditions, YAP is stabilized and associates with HIF-1α and PKM2 to drive PKM2 gene transcription and accelerate glycolysis [173]. In metastatic colorectal cancer, aberrant EGFR signaling induces PKM2 phosphorylation and YAP binding, upregulating GLUT3 and glycolytic enzymes to promote invasion [64]. Similarly, ErbB2 triggers PKM2-YAP complex formation and nuclear translocation to induce cancer stem-like cell properties [186]. By contrast, omentin-1 increases FBP-mediated PKM2 tetramerization to inhibit PKM2 dimerization, nuclear import, and YAP binding, promoting myofibroblasts’ lipogenic differentiation [174].

### Genes associated with lipid metabolism

nPKM2 influences lipid metabolism by binding and stabilizing nuclear SREBP-1a, thereby promoting the transcription of lipogenic genes such as fatty acid synthase (FASN) to support membrane biogenesis and energy storage [180]. Recent work in triple‐negative breast cancer (TNBC) further illustrates how nPKM2 can reprogram lineage and metabolism through epigenetic means. Zhang et al. showed that loss of PKM2-mediated glycolysis in TNBC cells triggers a compensatory switch toward fatty acid β‐oxidation (FAO), a process that requires increased carnitine import [176]. Mechanistically, PKM2 binds directly to the histone methyltransferase EZH2 and co-represses the carnitine transporter gene *SLC16A9* via H3K27 trimethylation. Inhibition or depletion of PKM2 disrupts EZH2 recruitment at the *SLC16A9* promoter, de-repressing its expression, boosting intracellular carnitine levels, and driving TNBC cells into an FAO-dependent, luminal-like state. Dual targeting of EZH2 and FAO in these cells induces synthetic lethality, highlighting how nPKM2’s nuclear interactions not only control glycolytic gene programs but also enforce epigenetic barriers that govern metabolic plasticity and lineage commitment in cancer.

### Genes associated with inflammatory responses

nPKM2 partners with NF-κB p65 under hypoxia via IGF-1/IGF-1R signaling to facilitate VEGF and angiogenesis [8], and it recruits p300 to p65, creating a platform for p300-mediated acetylation of NF-κB. This modification enhances the expression of IL-6, IL-8, and other inflammatory factors, thus promoting gastric cancer cell proliferation [40].

### Genes associated with anti-oxidative responses

nPKM2 modulates redox homeostasis by partnering with NRF2 [137]. Through this interaction, it upregulates antioxidant genes involved in glutathione synthesis and reactive oxygen species detoxification, thereby protecting cancer cells from oxidative stress [85, 137].

### Genes associated with cell cycle progression

In response to EGF, PKM2 translocates to the nucleus where it forms a ternary complex with β-catenin and c-Src. c-Src phosphorylates β-catenin at Y333, and this activated complex is recruited to the *CCND1* promoter. PKM2’s kinase activity modifies H3 T11, leading to HDAC3 removal from the promoter and facilitating H3K9 acetylation and cyclin D1 transcription [156, 157]. By contrast, HDAC8 deacetylates PKM2 at K62 to facilitate PKM2 transport into the nucleus and binds β-catenin, thereby promoting *CCND1* gene transcription and cell cycle progression [169]. nPKM2 also partners with Oct4 to regulate CCND1 and c-Myc, promoting cancer stem cell maintenance under stress conditions [157, 169, 186].

## nPKM2 as a protein kinase and an RNA-binding protein

Beyond its coactivator partnerships, nPKM2 exerts protein kinase activity and directly impacts oncogenic signaling and chromatin structure. nPKM2 phosphorylates STAT3 at Y705, enhancing STAT3’s transcriptional potency and promoting the expression of pro-inflammatory cytokines and proliferation-associated genes [158, 185]. Similarly, nPKM2 phosphorylates H3 at the T11 residue, inducing local chromatin relaxation that augments HIF-1α-dependent transcription of CCL2, promoting microglial polarization in peri-infarct and neuroinflammation [37]. Pharmacologic stabilization of PKM2’s tetrameric state by the small molecule PA-12 prevents the nuclear translocation of PKM2 and thus suppresses HIF-1α target gene expression in lung cancer cells [60].

In addition to its protein kinase function, nPKM2 has been shown to bind RNA directly. A recent study uncovered that PKM2 recognizes and binds folded RNA G-quadruplex (rG4) structures in pre-mRNAs, facilitating transcriptional elongation and upregulating EMT-related genes in breast cancer cells [1].

These findings underscore the multifaceted role of nPKM2 as both a gene programmer and metabolic modulator. By orchestrating the expression of key metabolic and oncogenic genes, nPKM2 supports tumor growth, adaptation to metabolic stress, and resistance to apoptosis, highlighting its potential as a therapeutic target in cancer.

## nPKM2 as a therapeutic target: blockers and inducers

As previously discussed, nPKM2 promotes oncogenic progression and represents an attractive therapeutic target in cancer treatment. Small molecules modulating PKM2 function can be categorized into two main groups based on their effects on its oligomeric state and nuclear localization: nPKM2 blockers and inducers. nPKM2 blockers act as anti-cancer agents by inhibiting PKM2 nuclear translocation, whereas nPKM2 inducers facilitate nuclear import and may promote tumorigenesis depending on the cellular context. This categorization is summarized in Table 5.

**Table 5:** Small molecule regulators of nPKM2

Molecule	PK activity	Tetramer	nPKM2 level	PDB	References
nPKM2 blockers					
Sulforaphane (Sfn)	–	Increase	Decrease		[9]
Coenzyme A (CoA)	–	Increase	Decrease		[24]
Salvianolic acid A (SAA)	–	Increase	Decrease		[188]
DASA-58	Increase	Increase	Decrease	3ME3	[3, 14]
TEPP-46 (ML-265)	Increase	Increase	Decrease	3U2Z	[3, 14]
Shikonin	Decrease	Decrease	Decrease		[22, 49]
Fructose 1,6-bisphosphate (FBP)	Increase	Increase		1T5A	[7, 34]
Serine	Increase	Increase	Decrease	4B2D	[20, 55]
Succinylaminoimidazolecarboxamide ribose-5′-phosphate (SAICAR)	Increase	–	Increase		[58, 59]
Polyphyllin II (PP2)	Increase	Increase	Decrease		[141]
CIAC001 (cannabidiol derivative)	Increase	Increase	Decrease		[55]
Quinoline sulfonamides (NZT)	Increase	Increase	Decrease		[63, 73]
7-Azaindole derivatives (compound 5, 6f)	–	Increase	Decrease		[79]
ZINC08383544	–	Increase	Decrease		[72]
nPKM2 inducers					
Scutellarin	Decrease	–	Increase		[160]
Compound 3 k	Decrease	Decrease	Increase		[42, 95]
Poly(ADP-ribose) (PAR)	–	–	Increase (Retention)		[71]
Phenylalanine	Decrease			4FXJ	[93]
Protocatechuic aldehyde (PCA)	Decrease	–	Increase		[142]
Ochratoxin A (OTA)	Decrease	–	Increase		[135]
Somatostatin analogue (TT-232)	–	–	Increase		[120]
Mechanism uncharacterized					
Asn/Asp	Increase	Increase	–	6V74/ 6V75	[94]
Val	Decrease	Decrease	–	6V76	[94]
Thyroid hormone (T3)	–	Decrease	–		[7]
Dimethylaminomicheliolide (DMAMCL, ACT001)	Increase	Increase	–		[67]
Compound 2	–	–	–	8G2E	[145]
Butyrate	Increase	Increase	–		[72]
Mannich base derived from lawsone (MB-6a)	Decrease	–	–		[107]

Mechanistically, nPKM2 blockers prevent nuclear import of PKM2 by stabilizing its tetrameric, enzymatically active state or by disrupting protein–protein interactions and PTMs essential for dimeric or monomeric formation. Natural products such as sulforaphane, bleomycin, vitamin B5 (pantothenate), quercetin, salvianolic acid A, kaempferol, and apigenin have demonstrated the ability to inhibit PKM2 nuclear translocation, effectively suppressing tumor growth and inflammatory signaling in various cancer and immune cell contexts [9, 18, 24, 76, 139, 140, 160, 181, 188].

To therapeutically target nPKM2, several synthetic small-molecule activators, including sulfonamide derivatives (DASA-58, 8 k/8b) and thieno[3,2-b]pyrrole[3,2-d]pyridazinone derivatives (TEPP-46), bind a well-defined allosteric pocket at the intersubunit **A**–**A** interface of PKM2, as revealed by high-resolution crystal structures [3, 6, 14, 79, 125]. This interaction locks PKM2 in its active tetrameric conformation, preventing the conformational changes required for nuclear import and thereby suppressing nPKM2-driven oncogenic programs.

Shikonin, a bioactive naphthoquinone compound derived from the roots of *Lithospermum erythrorhizon*, exhibits considerable therapeutic promise against cancer, inflammation, and wound healing. Shikonin inhibits tumor progression by multiple mechanisms, including suppression of cell proliferation and migration, induction of apoptosis, autophagy, necroptosis, and elevation of reactive oxygen species. It also activates anti-tumor immunity through modulation of signaling pathways such as PI3K/AKT/mTOR and MAPKs, as well as targeting various molecular regulators like Src, FAK, and RIP1/3 [41]. Shikonin is found to target PKM2 directly by inhibiting PKM2-mediated aerobic glycolysis, thus disrupting cancer cell metabolic reprogramming crucial for tumor growth and survival [168]. It prevents PKM2 nuclear translocation, further inhibiting its transcriptional regulation capabilities and suppressing tumor proliferation and progression [30, 164]. Structurally modified shikonin derivatives undergoing investigation demonstrate enhanced anticancer potency and reduced toxicity, indicating the therapeutic potential of targeting nPKM2 through natural and modified compounds [41, 150].

Conversely, nPKM2 inducers enhance PKM2’s nuclear functions. Natural agents such as scutellarin, ochratoxin A, the somatostatin analogue TT-232, and synthetic Compound 3 k have been shown to increase PKM2’s nuclear accumulation and coactivator activity [42, 120, 135, 160]. In addition, melatonin drives PKM2 into the nucleus of hippocampal neurons to partner with NRF2 and induce GPX4 expression, protecting against radiation-induced ferroptosis, while thymoquinone upregulates PKM2’s nuclear signaling via NF-κB, PI3K/AKT, and MAPK pathways to promote survival and inhibit apoptosis in pancreatic cancer cells [105, 182];

Targeting nPKM2 through modulators that stabilize its oligomeric state or influence nuclear localization provides a nuanced therapeutic approach for cancer treatment. Understanding the specific context-dependent effects and molecular mechanisms of these modulators, including their binding interactions, impact of PTMs, and their potential synergy with conventional cancer therapies, will be critical in the development of precision-targeted treatments exploiting nPKM2 in cancer and related metabolic diseases.

## Discussion and future perspectives

### nPKM2 as an opportunistic oncogene: signal-stimulated and context-dependent

This review highlights the pivotal role of nPKM2 as an oncoprotein that integrates metabolic reprogramming with transcriptional regulation, driving tumor growth and survival. It is an opportunistic oncogene in that it rises to the occasion when cancer cells face stressful conditions under the tumor microenvironment. The extracellular signals often induce their translocation. In its nuclear form, PKM2 functions as a co-transcriptional activator to upregulate genes involved in glycolysis, lipogenesis, and antioxidant defense, thereby sustaining the Warburg effect and enabling cancer cells to adapt to stress. There is considerable evidence that nPKM2 is the oncogenic component of PKM2: 1) nPKM2 transcriptionally activates genes involved in cancer metabolism, EMT, and drug resistance responses. 2) Blocking dimer formation or nPKM2 translocation often leads to attenuation of tumorigenic properties. 3) Cancer-prone mutations of PKM2 facilitate nuclear translocation. Indeed, in many cases, the nuclear PKM2 represents a minor fraction, with the level of cytoplasmic PKM2 remaining largely unchanged. Yet, there is a profound difference in the transformation phenotype. This is, however, similar to other oncogenic transformation events, where gain-of-function phosphorylated oncoproteins may represent only a small fraction of the total proteins, yet the effects are highly profound.

Although nPKM2 is best known for its pro-survival roles in cancer, it can, under certain conditions, act as a “double-edged sword,” sensitizing cells to stress or even promoting cell death. For example, in p53-mutant TNBC, Chk2 phosphorylates nPKM2 at S100, driving its nuclear export, reducing glycolytic flux, and suppressing vasculogenic mimicry, which collectively limit tumor aggressiveness [161]. Conversely, in cardiomyocytes, a small pool of PKM2 phosphorylated at S37 translocates to the nucleus, where it scaffolds transcription factors to protect GATA4/6 from caspase-1-mediated cleavage and recruits MDM2 to degrade pro-apoptotic p53. Loss of this nuclear pool, via TRIM35-mediated ubiquitination, destabilizes GATA4/6, elevates p53, and drives dilated cardiomyopathy in mice and humans, revealing a critical non-metabolic role for PKM2 in cardiac survival [84]. Moreover, the redox state of tetrameric PKM2 at C423 governs its regulation of p53: in high-oxidation tissues such as the heart, oxidized PKM2 suppresses p53-mediated apoptosis, whereas in the reduced, low-oxidation environment of tumors it enhances p53’s pro-apoptotic transcriptional program. Pharmacologic stabilization of the tetrameric form with TEPP-46 protects cardiomyocytes from doxorubicin-induced apoptosis while amplifying tumor cell death and regression in lung cancer models [111]. Thus, nPKM2’s impacts on cell fate are highly context-dependent on post-translational modifications, interacting partners, and redox milieu, which highlights the importance of understanding this plasticity when designing nPKM2-targeted therapies.

### nPKM2 as a therapeutic target: promises and problems

Given that non-malignant cells usually express PKM1 and likely do not need nPKM2 for Warburg effects or cancer metabolism, a promising therapeutic strategy is the development of nPKM2 blockers that stabilize PKM2 in its cytosolic tetrameric state, thus preventing its nuclear import and the subsequent activation of oncogenic transcription. Indeed, in breast cancer cell models, it was found that nPKM2 is critical for the survival of cancer cells, but not for non-transformed cells [186]. Currently available blocker agents include sulforaphane, bleomycin, vitamin B5, quercetin, and salvianolic acid A, along with synthetic compounds like TEPP-46, DASA-58, PA-12, and select 7-azaindole derivatives, which have been shown to effectively block nPKM2 formation and inhibit HIF-1α-mediated gene expression [3, 6, 14, 60, 79, 125]. Although these PKM2 activators (tetramer stabilizers) and nPKM2 blockers have demonstrated impressive anti-tumor activity in preclinical models, several hurdles must be cleared before first-in-human studies. First, locking PKM2 in a high-activity tetramer form can sustain the flux from PEP to pyruvate and deplete glycolytic intermediates needed for the biosynthesis of nucleotides, serine, and lipids, potentially starving tumors but also leading to compensatory up-regulation of the pentose phosphate pathway, serine synthesis, fatty acid oxidation, or glutaminolysis. Systematic metabolomic, flux-tracing, and redox-omics profiling of matched tumor and normal proliferative tissues will therefore be critical to uncover synthetic-lethal nodes and flag on-target toxicities early.

An alternative strategy is to directly block the nuclear translocation of PKM2. Although no peptide inhibitors of the PKM2-importin α5 interaction have been reported yet, there is a relevant precedent. SV40 large T-antigen-derived NLS peptides can competitively inhibit importin binding and prevent the nuclear entry of cargo proteins [61]. Similarly, a peptide could be designed to mimic the exon-10 NLS-encoded region of PKM2 and be evaluated for its ability to prevent PKM2’s nuclear translocation. Furthermore, detailed pharmacokinetic and selectivity profiles are necessary to ensure that tumors receive adequate exposure to the treatment while minimizing off-target toxicity.

Second, robust biomarkers are needed to identify patients whose cancers are “addicted” to nPKM2 and to confirm target engagement. Beyond immunohistochemical staining for nuclear PKM2 in tumor biopsies, circulating biomarkers, such as exosomal PKM2 [126] or its phospho-forms (p-S37, p-Y105) in plasma (Table 1), could provide minimally invasive readouts, while assays for nPKM2-driven epigenetic marks (e.g. H3-T11 phosphorylation or STAT3 phosphorylation) (Table 3) in core biopsies may offer complementary information on on-target activity.

Third, resistance and metastasis may emerge through compensatory adaptations; tumors can upregulate alternative transcriptional co-activators, rewire parallel metabolic circuits, or alter the expression or affinity of nuclear import machinery. To anticipate these escape mechanisms, systematic profiling of treated models using next-generation genomics, metabolomics, and single-cell transcriptomics will be essential [31]. Insights from such studies can then inform the design of rational combination therapies to circumvent or delay clinical challenges.

Early clinical trials are already underway. SYX-5219, a brain-penetrant PKM2 activator that is currently in Phase Ia for atopic dermatitis, serves as an example of safety optimization in non-oncology indications. Additionally, TP-1454, a potent PKM2 activator that stabilizes the enzyme in its cytosolic tetramer form, is being evaluated for safety and tolerability in a Phase I trial enrolling patients with advanced metastatic solid tumors.

Finally, because PKM2 serves homeostatic roles in normal proliferative tissues, chronic inhibition may carry unforeseen toxicities. A thorough off-target assessment and exploration of intermittent or conditional dosing schedules will help define a safe therapeutic window. Moreover, the context-dependent functions of nPKM2, in hypoxic glycolysis, antioxidant defense, or lipid anabolism, need to be modeled across diverse tumor microenvironments to ensure broad applicability. Addressing these challenges in parallel will be critical for successfully translating nPKM2 antagonists into clinical practice, optimizing both efficacy and tolerability.

Since the discovery of nPKM2 in 2012, we have come a long way in understanding its functions as a coactivator of transcription factors. We know that nPKM2’s protein kinase activity activates STAT3 and modifies histone H3 to increase acetylation of the nearby chromatin. Whether nPKM2 carries out additional coactivator functions such as increasing the affinity of transcription factors toward specific sites, shifting the binding spectrum of the transcription factors, or directly binding to DNA remains unclear. Also unclear is the detailed structure of the PKM2-transcription factor complex, especially those involving three components. Whether they truly form a ternary complex or are individual dimers remains to be established.

## Data Availability

No datasets were generated or analyzed during the current study.

## Abbreviations

PK: Pyruvate kinase
PKM2/1: Pyruvate kinase M2/M1
nPKM2: Nuclear PKM2
PEP: Phosphoenolpyruvate
PTMs: Post-translational modifications
FBP: Fructose-1,6-bisphosphate
NLS: Nuclear localization signal
JMJD5/KDM8: Lysine demethylase 8
DASA-58: Sulfonamide derivative
TEPP-46: Thieno[3,2-b]pyrrole[3,2-d]pyridazinone derivative
ERK1/2: Extracellular signal-regulated kinase 1/2
FGFR1/ALK: Fibroblast growth factor receptor 1/anaplastic lymphoma kinase
Syk: Spleen tyrosine kinase
PIN1: Peptidyl-prolyl cis-trans isomerase NIMA-interacting 1
PCAF: P300/CBP-associated factor
p300: Histone acetyltransferase p300
TIP60: Histone acetyltransferase TIP60
PIAS3: Protein inhibitor of activated STAT3
SUMO: Small ubiquitin-like modifier
PAR: Poly(ADP-ribose)
DDX39B: DExD-box helicase 39B
PDIA3: Protein disulfide-isomerase A3
EGFRvIII: Epidermal growth factor receptor variant III
ErbB2/HER2: Receptor tyrosine-protein kinase erbB-2/Human epidermal growth factor receptor 2
SphK1: Sphingosine kinase 1
KHK: Ketohexokinase
GSK3β: Glycogen synthase kinase-3 beta
ATM: Ataxia telangiectasia-mutated kinase
AKT: Serine/threonine kinase AKT
Cdc25A: Cell division cycle 25A
c-Myc: Cellular myelocytomatosis oncogene
PPP1R26: Protein phosphatase 1 regulatory subunit 26
ATP6V0C: Vacuolar H+-adenosine triphosphatase subunit V0C
eEF2K: Eukaryotic elongation factor-2 kinase
SAE1: SUMO-activating enzyme 1
UBA2: Ubiquitin-like modifier activating enzyme 2
CHAC1: Glutathione-specific gamma-glutamylcyclotransferase 1
ESM1: Endothelial cell-specific molecule 1
GTPBP4: Guanosine triphosphate binding protein 4
TRIM33: Ectodermin homolog and tripartite motif-containing 33
PSMD14: 26S proteasome non-ATPase regulatory subunit 14
EGF: Epidermal growth factor
SIRT1/2/5/6: Sirtuin 1/2/5/6
PSAT1: Phosphoserine aminotransferase 1
HDAC3/8: Histone deacetylase 3/8
Chk2: Checkpoint kinase 2
EXT1: Exostosin-1
JOSD2: Josephin domain-containing 2
SHP-1: Src homology region 2 domain-containing phosphatase 1
LA: Lambertianic acid
SAA: Salvianolic acid A
ART: Artemisinin
TSC22D2: Transforming growth factor β-stimulated clone 22 domain family, member 2
MNX1-AS1: MNX1 antisense RNA 1
HIFAL: HIF-1α anti-sense lncRNA
PHD3: Prolyl hydroxylase 3
AC020978: LncRNA-AC020978
HIF-1α: Hypoxia inducible factor 1α
FEZF1-AS1: FEZF1 antisense RNA 1
BLM: Bleomycin
IGF-1: Insulin-like growth factor 1
IGF-1R: Insulin-like growth factor 1 receptor
HGF: Hepatocyte growth factor
TGFβ: Transforming growth factor beta
EMT: Epithelial–mesenchymal transition
PD-L1: Programmed cell death ligand 1
IL-3/6/8: Interleukin 3/6/8
Jak2: Janus kinase 2
SHMT2: Serine hydroxymethyltransferase 2
AMPK: AMP-activated protein kinase
PCBs: Polychlorinated biphenyls
CCl_4_: Carbon tetrachloride
CCL2: C-C motif chemokine ligand 2
HRE: Hypoxia response element
LDHA: Lactate dehydrogenase A
GLUT1/3: Glucose transporter type 1/3
PFKFB3: 6-Phosphofructo-2-kinase/Fructose-2,6-biphosphatase
YAP: Yes-associated protein
SREBP-1a: Sterol regulatory element-binding protein 1a
FASN: Fatty acid synthase
TNBC: Triple‐negative breast cancer
FAO: Fatty acid β‐oxidation
EZH2: Enhancer of zeste homolog 2
SLC16A9: Solute carrier family 16 member 9
NF-κB/p65: Nuclear factor kappa-light-chain-enhancer of activated B cells
VEGF: Vascular endothelial growth factor
c-Src: Proto-oncogene tyrosine-protein kinase Src
CCND1: Cyclin D1
Oct4: Octamer-binding transcription factor 4
STAT3: Signal transducer and activator of transcription 3
rG4: RNA G-quadruplex
PI3K: Phosphatidylinositol 3-kinase
mTOR: Mammalian target of rapamycin
MAPK: Mitogen-activated protein kinase
FAK: Focal adhesion kinase
RIP1/3: Receptor-interacting protein 1/3
p53: Tumor protein p53
GATA4/6: GATA binding protein 4/6
MDM2: Murine double minute 2
TRIM35: Tripartite motif containing 35
SV40: Simian vacuolating virus 40
